# Microbiological components in mainstream and sidestream cigarette smoke

**DOI:** 10.1186/1617-9625-10-13

**Published:** 2012-08-16

**Authors:** Lennart Larsson, Christina Pehrson, Tenzin Dechen, Mardi Crane-Godreau

**Affiliations:** 1 Dept of Laboratory Medicine, Lund University, Lund, Sweden; 2 Dept of Microbiology & Immunology, Geisel School of Medicine at Dartmouth, Lebanon, NH, USA

**Keywords:** Lipopolysaccharide, Ergosterol, Tobacco, Cigarette smoke

## Abstract

**Background:**

Research has shown that tobacco smoke contains substances of microbiological origin such as ergosterol (a fungal membrane lipid) and lipopolysaccharide (LPS) (in the outer membrane of Gram-negative bacteria). The aim of the present study was to compare the amounts of ergosterol and LPS in the tobacco and mainstream (MS) and sidestream (SS) smoke of some popular US cigarettes.

**Methods:**

We measured LPS 3-hydroxy fatty acids and fungal biomass biomarker ergosterol in the tobacco and smoke from cigarettes of 11 popular brands purchased in the US. University of Kentucky reference cigarettes were also included for comparison.

**Results:**

The cigarette tobacco of the different brands contained 6.88-16.17 (mean 10.64) pmol LPS and 8.27-21.00 (mean 14.05) ng ergosterol/mg. There was a direct correlation between the amounts of ergosterol and LPS in cigarette tobacco and in MS smoke collected using continuous suction; the MS smoke contained 3.65-8.23% (ergosterol) and 10.02-20.13% (LPS) of the amounts in the tobacco. Corresponding percentages were 0.30-0.82% (ergosterol) and 0.42-1.10% (LPS) for SS smoke collected without any ongoing suction, and 2.18% and 2.56% for MS smoke collected from eight two-second puffs.

**Conclusions:**

Tobacco smoke is a bioaerosol likely to contain a wide range of potentially harmful bacterial and fungal components.

## Background

Tobacco smoke contains several thousands of chemicals and most of them are formed during the burning of the tobacco [1]. Other chemicals in the smoke may be present in the tobacco itself, surviving the combustion during the smoking. Examples are compounds from microorganisms that naturally colonize tobacco leaves. That tobacco is rich in both bacteria and in fungi has been known for decades [2], but it was not until modern molecular biology methods became available, such as 16S rRNA sequence analysis, that a rich diversity of the microbes in tobacco was revealed. Thus, a large number of species of both Gram-positive and Gram-negative bacteria and of molds have been identified [3,4]. Hasday et al. [5] found that cigarette smoke contains biologically active endotoxin; this was the first time that a specific group of bacterial toxins were proven to be present in tobacco smoke. Larsson et al. [6,7] used gas chromatography-tandem mass spectrometry (GC-MSMS) to demonstrate the presence of lipopolysaccharide (LPS), i.e. the endotoxin molecule of the outer membrane in all Gram-negative bacteria, in smoke. The LPS concentrations in indoor air were directly proportional to the number of cigarettes smoked during a given time interval [8]. Ergosterol, a fungal membrane lipid, was also identified, both in the tobacco and the smoke. A positive relationship was found between the amounts of LPS and ergosterol in the tobacco of a studied cigarette and the amounts of these substances in mainstream smoke [7]. The concentrations of the microbiological compounds in the tobacco of some local brands of Vietnamese and Chinese cigarettes were much lower than in the tobacco of international brands of cigarettes purchased in different European and Asian countries [7]. The reason for this difference is unknown; however, both pesticides and fungicides are known to be common on tobacco plantations.

The studies published so far on ergosterol and LPS in tobacco smoke have utilized a home-made sampling device for collecting mainstream (MS) smoke; sidestream (SS) smoke has to date not been analyzed for these compounds. The aim of the present study was to measure LPS and ergosterol in the tobacco as well as in MS and SS smoke of several brands of popular cigarettes purchased in the US. LPS was measured by determining the number of moles of 3-hydroxy fatty acids (3-OH FAs) of 10–18 carbon chain lengths [6]. The smoke samples were generated by adapting a Teague TE-10, a standard research cigarette smoke generating device.

## Methods

### Cigarette tobacco and smoke samples

Packs of cigarettes of 11 US brands (Camel Turkish Royal, Marlboro Red, Marlboro Light, Marlboro Menthol, Pall Mall, Winston, Newport Menthol, Kool, Parliament Lights, Basic Full Flavor, and Liggett Select Full Flavor) were purchased at a store in Boston MA and kept in a freezer (−20 C) until use. University of Kentucky 3R4F research cigarettes were used for reference.

Tobacco from one cigarette of each of the 11 brands plus one University of Kentucky reference cigarette was studied. In addition, samples of MS and SS smoke of cigarettes of six brands (Camel Turkish Royal, Pall Mall, Newport Menthol, Kool, Parliament Lights, and Liggett Select Full Flavor), plus University of Kentucky reference cigarettes, were used. The smoke was generated using a smoking machine and collected on Pall Life Sciences, PallFlex Membrane Filters (PMMF, EmFab TX40H120-WW). In each series of experiments a filter that had not been exposed to the smoke was used as a negative control.

The smoking apparatus used was a Teague Enterprises TE-10 smoking system equipped with two exposure chambers [9,10]. The smoking device is microprocessor controlled and produces both MS and SS smoke, or SS smoke alone. Smoke is drawn from the combustion chamber into a mixing chamber and then into exposure chambers. Valves control airflow and permit purging smoke from within the mixing and exposure chambers into dedicated ducting that conducts all purged smoke outside the building. In the SS smoke experiments all smoke was directed into the upper chamber. In the MS smoke experiments, the smoke was drawn through a lit cigarette into a Buchner flask. Although one cigarette burns out in less than one min, 6 min suction was used to assure that the flask was fully evacuated of smoke. SS smoke was collected from lit cigarettes without any ongoing suction. The suction on the (larger) chamber was run for 12 min followed by a "blow out" to prevent any remaining smoke in the collection chamber.

In three experiments smoke from lit Marlboro light cigarettes was collected using 8 puffs (2 seconds/puff) per cigarette in order to estimate the smoke concentrations of LPS and ergosterol under realistic smoking conditions. Only MS smoke, from one cigarette/experiment, was collected in a flask and then drawn into the filter during 6 min.

### Sample preparation and analysis

The tobacco samples (of one whole cigarette each) were weighed and, just as the PMMF filter samples, transferred to glass test tubes equipped with Teflon-lined screw caps. One set of samples was prepared for analysis by GC-MSMS of 3-OH FAs following acid methanolysis and silylation and a separate set of samples was prepared for analysis of ergosterol following alkaline hydrolysis and silylation, as described [11]. The entire methanolysate and hydrolysate samples of the filters and a 1/100th fraction of the methanolysate and hydrolysate samples of the tobacco were subjected to the subsequent preparatory steps before being analysed. The amounts of LPS were calculated by summarizing the number of moles of the 3-OH FAs and dividing by four, assuming that one mole of LPS contains four moles of 3-OH FAs [11].

### Statistical analysis

The Spearman and Pearson tests were used for studying correlations between the amounts of LPS and ergosterol in the cigarette tobacco with the amounts in MS and SS smoke.

## Results

### LPS and ergosterol in cigarette tobacco

The cigarettes contained each approximately 700 mg of tobacco. One mg of tobacco contained 6.88 – 16.17 (mean 10.64, SD 1.91) pmol LPS and 8.27-21.0 (mean 14.05, SD 4.25) ng ergosterol (Table 1).

**Table 1:** LPS and ergosterol in cigarette tobacco (N = 1)

**Cigarette**	**Erg ng/mg**	**LPS pmol/mg**
Camel Turkish Royal	9.12	6.88
Marlboro Red	15.99	10.77
Marlboro Light	13.65	11.15
Marlboro Menthol	14.70	7.84
Pall Mall	11.29	8.83
Winston	14.04	12.83
Newport Menthol	8.27	10.67
Kool	13.23	16.17
Parliament Lights	15.10	13.60
Basic Full Flavor	13.61	11.10
Liggett Select Full Flavor	18.56	8.80
University of Kentucky	21.00	9.00

### LPS and ergosterol in tobacco smoke

Cigarettes of six brands containing relatively high and relatively low or medium concentrations of the microbiological compounds, respectively, plus one University of Kentucky cigarette, were selected for analysis of MS and SS smoke. MS smoke from one cigarette produced by continuous suction contained 384–725 ng ergosterol and 823–1504 pmol LPS corresponding to 3.65 – 8.23% and 10.02 - 20.13%, respectively, of the amounts in the tobacco per cigarette of a given brand. By comparison, SS smoke collected from one cigarette without any ongoing suction contained 17 – 78 ng ergosterol and 41–71 pmol LPS corresponding to 0.30-0.82% and 0.42-1.10%, respectively, of the amounts in the tobacco per cigarette of a given brand (Table 2).

**Table 2:** LPS and ergosterol in the cigarette tobacco and smoke; percentages of LPS and ergosterol in the smoke in relation to the tobacco (N = 1)

**Cigarette**	**Erg ng/cig**	**%**	**LPS pmol/cig**	**%**
**Newport Menthol**				
Tobacco	5790		7470	
Sidestream	17	0.30	51	0.68
Mainstream	384	6.63	1504	20.13
**Camel Turkish Royal**				
Tobacco	6380		4820	
Sidestream	52	0.82	51	1.06
Mainstream	525	8.23	823	17.07
**Pall Mall**				
Tobacco	7900		6180	
Sidestream	61	0.77	68	1.10
Mainstream	457	5.78	1130	18.28
**Kool**				
Tobacco	9260		11320	
Sidestream	40	0.43	48	0.42
Mainstream	429	4.63	1134	10.02
**Parliament Lights**				
Tobacco	10570		9520	
Sidestream	49	0.46	41	0.43
Mainstream	488	4.62	1363	14.32
**Liggett Select Full Flavor**				
Tobacco	12990		6160	
Sidestream	41	0.32	42	0.68
Mainstream	474	3.65	1044	16.95
**University of Kentucky**				
Tobacco	15120		6480	
Sidestream	78	0.52	71	1.10
Mainstream	725	4.79	1130	17.44

The proportions (percentages) of ergosterol and LPS in the SS smoke samples (collected without any suction) in relation to the MS smoke samples (collected by continuous suction) were in all cases higher for ergosterol (mean 9.47%) than for LPS (mean 4.73%) (Table 3).

**Table 3:** Percentage of ergosterol and LPS in sidestream smoke in relation to mainstream smoke

**Cigarette**	**Erg %**	**LPS %**
**Newport Menthol**	4.45	3.36
**Camel Turkish Royal**	9.90	6.18
**Pall Mall**	13.3	6.01
**Kool**	9.30	4.26
**Parliament Lights**	10.00	3.00
**Liggett Select Full Flavor**	8.57	3.99
**University of Kentucky**	10.86	6.30

MS smoke collected by the 8-puff samplings contained on average 0.20 nmol LPS and 218 ng ergosterol per filter, thus 2.56% and 2.18%, respectively, of the amounts in the cigarette tobacco.

Significant correlations were found between the amounts of LPS in cigarette tobacco and in MS smoke (Spearman, *P* = 0.016), between ergosterol in cigarette tobacco and MS smoke (Pearson, *P* = 0.075), and between ergosterol in SS and MS smoke (Pearson, *P* = 0.015; Spearman, *P* = 0.036). The MS samples had been collected using continuous suction and the SS samples had been collected without any suction as described above.

## Discussion

Cigarette smoke contains thousands of chemicals many of which pose a serious health risk. These chemicals may be formed during the burning of the tobacco or may be present in the tobacco itself. Among the latter group of compounds are pesticides and other intact agrochemicals, menthol, flavorants etc. [12]. In recent years it has been demonstrated that also products from microorganisms, such as from bacteria and molds that are ubiquitous in tobacco, may be found in the smoke. Thus, Edinboro et al. [13] identified aflatoxin B1 in SS cigarette smoke by using immunoaffinity column extraction coupled with liquid chromatography/mass spectrometry, and Hasday et al. [5] used the *Limulus* test to reveal endotoxin in cigarette smoke. The reaction from the tobacco industry following the reports in scientific press on the presence of biologically active endotoxin in smoke was reviewed recently [14].

Tobacco is an agricultural product and as such contains a myriad of colonising bacteria and fungi. In a previous study we found that the microbial load in a cigarette mostly originates from colonization of the tobacco leaves on the field [7]. Among the microbes found in tobacco, identified by using molecular biological methods and/or microscopy, culturing, and conventional biochemical tests, are for example *Acinetobacter, Bacillus, Burkholderia, Clostridium, Klebsiella, PseudomonasSerratia, CampylobacterEnterococcusProteusStaphylococcusPantoea,* a large range of mesophilic and thermophilic bacteria, and fungi such as for example *Aspergillus*[4,7,15,16]. Bacteria in cigarettes were speculated to be associated with an outbreak of severe pneumonitis in military personnel [17]; bacteria growing on tobacco flakes have been hypothesized as representing a health risk to the smoker [18].

We introduced chemical marker analysis as a new concept for assessing the microbiological contents of tobacco and smoke [6,7]. With this technology the total bacterial biomass in a sample is determined by measuring the amounts of muramic acid, an amino sugar present exclusively in peptidoglycan. Analogously, ergosterol, a specific fungal membrane lipid, is used to quantify fungal biomass, and 3-OH FAs of 10 – 18 carbon chain lengths are used to quantify LPS and/or the biomass of Gram-negative bacteria [11]. The amounts of ergosterol and LPS found in the tobacco samples here studied are in general agreement with those found previously in cigarettes of international brands purchased in countries in Europe and Asia. At the same time, these amounts are much higher - up to 20-fold - than in some local cigarettes purchased in China, Korea, and Vietnam [7]. In the present study we did not include muramic acid as an analyte since the amounts in MS and second hand smoke are so low that they are barely detectable [7].

MS smoke collected by eight two-second puffs contained 2-3% of the amounts of LPS and ergosterol present in the tobacco of one cigarette. These results are in general agreement with those found previously where smoke had been collected by using home-made sampling with a gas-tight syringe [7]. Smoke produced by eight two-second puffs most closely resembles smoke that would occur in a closed space under normal cigarette usage. That is, the number of puffs taken on the cigarette and the length and volume of the puffs are designed to simulate human smoking behaviour.

Use of continuous suction for collecting MS smoke gave a circa 10 times higher smoke/tobacco proportions of LPS and ergosterol as compared with the puffing. The reason for the consistently higher smoke/tobacco proportion of LPS than of ergosterol found in MS smoke is unknown; however, LPS is known to be a heat-stable molecule. The amounts of ergosterol and LPS were much lower - by a factor of approximately 10 (ergosterol) and 20 (LPS), respectively - in SS smoke than in MS smoke. Indeed, microbiological compounds appearing in MS smoke may have been “distilled” through the cigarette during the smoking thus not being exposed to as high temperatures (thereby avoiding extensive thermal degradation) as at the tip of the burning cigarette, resulting in SS smoke. Notably, SS smoke is regarded as being more toxic than MS smoke [19] probably resulting from production of toxic components at very high temperatures.

We showed previously that adding gram-negative bacteria to a cigarette resulting in an 8-fold increase in LPS in the tobacco gave a 4-fold increase in MS smoke. Corresponding increases of ergosterol after fungi had been added to the tobacco were 15-fold (in tobacco) and 9-fold (in MS smoke) [7]. Significant correlations between the amounts of LPS and ergosterol in the cigarette tobacco and in MS smoke were also found in the present study. Taken together, these studies strongly indicate that the 3-OH FAs and ergosterol found in smoke stem from bacteria and fungi in the tobacco.

Microorganisms may not be evenly distributed in cigarette tobacco and this may result in difficulties in achieving reproducible analysis results. Analysis of SS smoke from two different University of Kentucky cigarettes revealed 72 and 84 ng ergosterol and 76 and 67 pmoles LPS, respectively (data not shown). Since tobacco from only a single cigarette of each brand was analysed the results of the present study cannot be used to compare different brands. But, the study demonstrates e.g. that a single cigarette of a popular brand, purchased in the US, may contain bacteria and fungi enough to produce 218 ng ergosterol and 0.20 nmoles of LPS at an average smoking behavior, with eight two-second puffs per cigarette. Notably, assuming an average molecular weight of environmental LPS of 8000, 0.2 nmoles of LPS corresponds to 1600 ng. We showed previously that smoking 2–12 cigarettes over a 5-h period resulted in 4–63 times increased concentrations of LPS in indoor air [8]. Cigarette smoke is known to induce inflammation of the lung [20]; further research is required to identify a possible role of the microbiological compounds in smoke in evoking inflammation. It is intriguing that such symptoms in the airways that are common among smokers are also common among nonsmokers with occupational exposure to bioaerosols [21,22].

## Conclusions

Cigarette tobacco contains large amounts of substances of microbiological origin. The compounds focused at in the present study, viz. LPS and ergosterol, should largely be viewed as markers of the plethora of microbial components present. We showed that MS smoke produced using a normal smoking behavior may contain more than 2% of the amounts of ergosterol and LPS that are present in the cigarette tobacco. Public awareness that cigarette smoke exposure entails inhaling toxic and inflammatory microbial compounds may help individuals make informed choices with respect to smoking.

## Competing interests

The authors declare that they have no competing interests.

## Authors’ contributions

LL was responsible for the over-all study design and did the main writing. CP made the LPS analyses. MC-G was responsible for production of smoke samples and contributed to the experimental design. TD assisted in the production of the smoke samples. All authors read and approved the final manuscript.

## References

[B1] Borgerding M Klus H Analysis of complex mixtures—cigarette smoke Exp Toxicology Pathol 2005 57 1 43 73 10.1016/j.etp.2005.05.010 16092717

[B2] Welty RE Fungi isolated from flue-cured tobacco sold in Southeast United States, 1968–1970 Appl Microbiol 1972 24 3 518 520 4627970 10.1128/am.24.3.518-520.1972 PMC376555

[B3] Huang J Yang J Duan Y Gu W Gong X Zhe W Su C Zhang KQ Bacterial diversities on unaged and aging flue-cured tobacco leaves estimated by 16S rRNA sequence analysis Appl Microbiol Biotechnol 2010 88 553 562 10.1007/s00253-010-2763-4 20645083

[B4] Sapkota AR Berger S Vogel TM Human pathogens abundant in the bacterial metagenome of cigarettes Environ Health Perspect 2010 118 3 351 356 20064769 10.1289/ehp.0901201 PMC2854762

[B5] Hasday JD Bascom R Costa JJ Fitzgerald T Dubin W Bacterial endotoxin is an active component of cigarette smoke Chest 1999 115 829 835 10.1378/chest.115.3.829 10084499

[B6] Larsson L Szponar B Pehrson C Tobacco smoking increases dramatically air concentrations of endotoxin Indoor Air 2004 14 421 424 10.1111/j.1600-0668.2004.00290.x 15500635

[B7] Larsson L Szponar B Ridha B Pehrson C Dutkiewicz J Krysinska-Traczyk E Sitkowska J Identification of bacterial and fungal components in tobacco and tobacco smoke Tob Induc Dis 2008 4 4 10.1186/1617-9625-4-4 PMC2556030 18822161

[B8] Sebastian A Pehrson C Larsson L Elevated concentrations of endotoxin in indoor air due to cigarette smoking J Environ Monit 2006 8 519 522 10.1039/b600706f 16688352

[B9] Teague SV Pinkerton KE Goldsmith M Gebremichael A Chang S Jenkins RA Moneyhun JH Sidestream smoke generation and exposure system for environmental tobacco smoke studies Inhal Toxicol 1994 6 79 93 10.3109/08958379409029697

[B10] Shang S Ordway D Henao-Tamayo M Bai X Oberley-Deegan R Shanley C Orme IM Case S Minor M Ackart D Hascall-Dove L Ovrutsky AR Kandasamy P Voelker DR Lambert C Freed BM Iseman MD Basaraba RJ Chan ED Cigarette smoke increases susceptibility to tuberculosis – evidence from in vivo and in vitro models J Infect Dis 2011 203 1240 1248 10.1093/infdis/jir009 21357942

[B11] Sebastian A Larsson L Characterisation of the microbial community in indoor environments: a chemical-analytical approach Appl Environ Microbiol 2003 69 3103 3109 10.1128/AEM.69.6.3103-3109.2003 12788704 PMC161488

[B12] Pauly JK Paszkiewicz G Cigarette smoke, bacteria, mold, microbial toxins, and chronic lung inflammation J Oncol 2011 2011 819129 21772847 10.1155/2011/819129 PMC3136185

[B13] Edinboro LE Karnes HT Determination of aflatoxin B1 in sidestream cigarette smoke by immunoaffinity column extraction coupled with liquid chromatography/mass spectrometry J Chromatogr A 2005 1083 127 132 10.1016/j.chroma.2005.06.032 16078698

[B14] Barnes RL Glantz SA Endotoxins in tobacco smoke: shifting tobacco industry positions Nicotine Tob Res 2007 9 10 995 1004 10.1080/14622200701488392 17852769

[B15] Papavassiliou J Piperakis G Marcelou-Kinti U Mycological flora of cigarettes Mycopathology Mycology Applied 1971 44 2 117 120 10.1007/BF02051879 5103523

[B16] Zhao M Wang B Li F Qiu L Li F Wang S Cui J Analysis of bacterial communities on aging flue-cured tobacco leaves by 16S rDNA PCR-DGGE technology Appl Microbiol Biotechnol 2007 73 6 1435 1440 10.1007/s00253-006-0625-x 17043820

[B17] Rooney AP Swezey JL Wicklow DT McAtee MJ Bacterial species diversity in cigarettes linked to an investigation of severe pneumonitis in U.S. military personnel deployed in Operation Iraqi Freedom Curr Microbiol 2005 51 1 46 52 10.1007/s00284-005-4491-z 15942700

[B18] Pauly JL Waight JD Paszkiewicz GM Tobacco flakes on cigarette filters grow bacteria: a potential health risk to the smoker? Tob Control 2008 17 1 49 52 10.1136/tc.2007.022772 18768459

[B19] Schick S Glantz S Philip Morris toxicological experiments with fresh sidestream smoke: more toxic than mainstream smoke Tob Control 2005 14 396 404 10.1136/tc.2005.011288 16319363 PMC1748121

[B20] Kulkarni GS Nadkarni PP Cerreta JM Ma S Cantor JO Short-term cigarette smoke exposure potentiates endotoxin-induced pulmonary inflammation Exp Lung Res 2007 33 1 1 13 10.1080/01902140601112957 17364908

[B21] Schwartz DA Thorne PS Yagla SJ Burmeister LF Olenchock SA Watt JL Quinn TJ The role of endotoxin in grain dust-induced lung disease Am J Respir Crit Care Med 1995 152 603 608 7633714 10.1164/ajrccm.152.2.7633714

[B22] Heldal KK Halstensen AS Thorn J Eduard W Halstensen TS Airway inflammation in waste handlers exposed to bioaerosols assessed by induced sputum Eur Respir J 2003 21 641 645 10.1183/09031936.03.00059702 12762350

